# Viewing conditions predict evolutionary diversity in avian plumage colour

**DOI:** 10.1098/rspb.2024.1728

**Published:** 2025-04-09

**Authors:** Jamie Dunning, Catherine Sheard, John A. Endler

**Affiliations:** ^1^ Department of Life Sciences, Imperial College London, London, UK; ^2^ Faculty of Biological Sciences, University of Leeds, Leeds LS2 3AA, UK; ^3^ Palaeobiology Research Group, University of Bristol, Bristol BS8 1TQ, UK; ^4^ School of Biological Sciences, University of Aberdeen, Aberdeen AB24 2TZ, UK; ^5^ Life and Environmental Sciences, Deakin University, Waurn Ponds, VIC 3216, Australia; ^6^ Department of Zoology and Ecology, James Cook University, Smithfield, Cairns, QLD 4870, Australia

**Keywords:** light environment, viewing conditions, habitat effects, plumage colour, spectrophotometry, bird plumage

## Abstract

Animals communicate using multiple sensory channels, including via vision. The colourful plumage of birds is a model system to study visual communication, having evolved through a complex interplay of processes, acting not only on the ability of a plumage patch to convey information, but also in response to physiological and environmental factors. Although much research on inter-specific variation in bird plumage has concentrated on sexual selection, much less has considered the role of non-sexual selection and how it is affected by the joint effects of avian viewing conditions and receiver vision. Here, we combined a taxonomically diverse database of avian plumage reflectance measurements with bird vision models, habitat and behavioural data to test the effect of three factors that affect viewing conditions—habitat openness, migratory preference and diel activity—on avian plumage contrast, accounting for shared evolutionary history and variation in avian visual systems. We find that habitat structure and migratory preference predicted plumage visual contrast, especially for females. Our study therefore demonstrates the important role of non-sexually selected traits, viewing conditions and bird vision, in shaping avian plumage contrast.

## 1. Introduction

The plumage of birds is a model system to study the evolution of visual communication because it is highly diverse compared with visual communication channels in other tetrapod clades [1–6]. Multiple interacting social and ecological processes generate and maintain this variation and, thus, present a challenge to understanding the evolution of avian signals more broadly [7–10]. For example, while many selective pressures act on plumage as a means of information transfer [10–12], the evolution of avian coloration is also affected by demands for thermoregulation [13–16] protection from abrasion [17–20] and bacterial degradation [21].

For successful visual communication, local light conditions, and even the direction of view are potentially very important [11,22–25]. Factors affecting these viewing conditions include behavioural traits like diel activity (the timing of activity across a day/night cycle) and fine-scale habitat use (e.g. where dense habitats are darker and greener than open ones [22], as well as the visual perception of the observer. For example, in darker environments such as dense forests or nocturnal settings, less intense colours (i.e. less reflective and less saturated colours with lower chroma) are harder to detect [26], while avian plumage patterns in the ultraviolet (UV) spectra will only be visible to species with UV sensitivity [26–28] and in environments with available UV light [22,23]. Assuming selection favours communication signals that maximize information relative to background noise [22,26], this ecological and behavioural context would thus be expected to shape interspecific plumage variation. These principles are also true for non-bird taxa, for example, in the evolution of pelage (fur) colour of many mammals [29–33].

Recent intraspecific studies have demonstrated the empirical basis for the importance of environmental viewing conditions as a selection pressure on avian plumage. For example, the rump colour of the Eastern yellow robin *Eopsaltria australis* is associated with regional vegetation density in Australia [34]. In the dichromatic red-capped plover *Charadrius ruficapillus*, brightly coloured males incubating the nest during daylight were found to attract predators at a higher rate than the less conspicuous females; but not when incubating at night [35]. Furthermore, many arboreal species balance the need to signal to conspecifics against predation risk by maintaining plumages that are cryptic in the context of forested light environments, for example the green and yellow plumages of wire-tailed manakin *Pipra filicauda* [36,37] and neotropical tanagers Thraupidae [38,39] iridescent patches in the plumage of hummingbirds Trochilidae [40] and the brown plumage of woodcreepers Furnariidae [41]. Nocturnal species may represent an extreme example of using light environments to maintain this balance; for example, super-white plumage patches may have evolved in response to a need for signalling in low-light environments [42,43], such as the extremely bright white tips of the Eurasian woodcock, *Scolopax rusticola* [43]. These effects can also be observed in smaller-scale comparative studies, for example the cone (photoreceptor) opsin gene expression in new world warblers, Parulidae sp., varies with sex but also, with species habitat preferences [23]. This is likely to alter their perception of visual signals in response to light environment and suggests that sexual dimorphism matches the classical prediction of coevolution of female preferences (opsins) and male traits (plumage [23]). That is, visual systems and visual signals are subject to both sexual and non-sexual selection.

Despite this evidence, that interspecific variation in bird plumage relates to viewing conditions (as in [37]), the majority of work on avian coloration has concentrated on sexual selection (e.g. [3,5,10]). Other behaviours, such as non-reproductive social behaviours or predator avoidance, may also play a role in structuring the variation of avian colour diversity. For example, social cohesion, including flocking and migration, depends upon efficient communication among members [44–46], and therefore highly visible plumage components are expected to co-occur with flock cohesion.

To investigate the broad-scale relationship between avian coloration and both social and ecological viewing conditions, we used a large database of bird plumage reflectance spectra, compiled from multiple sources, and applied a sensory ecology approach to investigate how key measures of avian visual conditions structure the diversity of avian plumage coloration. Specifically, we asked if differences in colour within individuals (‘visual contrast’) are predicted by three traits, (i) habitat density, (ii) the pattern of activity over a day/night cycle, hereafter called diel activity, and (iii) migration, under the hypothesis that intraspecific visual communication favours high visual contrast as estimated by avian colour vision models, ambient light and plumage reflectance spectra.

We tested three predictions based upon the assumption that more efficient intraspecific communication arises from higher visibility: (i) darker habitats (higher density [22]) should be associated with more contrasting coloration [10,22,47]; (ii) diel activity (daily activity times) will correlate with plumage contrast, with more achromatic contrast in nocturnal and perhaps crepuscular species compared to diurnal species, as in Dunning *et al*. [43]; and (iii) species which migrate or otherwise form coherent groups should show higher contrast than those which are more sedentary because higher visibility makes coherent movements easier in flight and on the ground. Further, migratory species may have been associated with greater sexual selection driven by less time to mate [48]. Migration occurs at all times of day but more commonly at night [49] nocturnal or crepuscular times would particularly favour high achromatic contrast. By day at rest, migratory species will experience multiple visual conditions (which might also favour higher contrast) but also have to contend with predation while they rest, which may favour reduced contrast by day. The contrast in migrants may thus represent a compromise between differing diurnal and nocturnal selective factors [50]. We tested these predictions in the two sexes separately as there is now ample evidence that the coloration of both sexes is naturally and sexually selected [51,52] and can vary independently from each other[8,53–55].

We used a phylogenetic comparative method (e.g. [9,56]) using generalized linear mixed models (GLMMs) with a Bayesian Markov chain Monte Carlo (MCMC) extension [57] to explore the relationships between plumage contrast, bird vision, light environments and behaviour. These relationships highlight the potential role of non-sexual behavioural factors in the evolution of avian plumage, as well as the importance of including the evolutionary and environmental perspectives of both the signaller and the receiver.

## 2. Material and methods

### (a) Plumage traits

We accessed reflection data from published and unpublished sources (including [38,43,58–66]), which have been compiled into a single database, BirdColorBase, by Thanh-Lan Gluckman and Peter Dunn [67].

Spectra were in the form of calibrated reflectance every 2 nm from 300 to 700 nm collected from 2610 species, using similar methods and equipment, representing 35 orders and 170 families and 984 genera. On average, there were 6.8 ± 2.8 (mean ± s.d.) birds scanned per species and 6.2 ± 2.6 different plumage patches scanned per bird per species. The database contains 352155 spectra of which 174794 are from adult males, 161952 are from adult females and 15409 are from individuals with unrecorded sex/maturity. Given the high rates of sexual dichromatism and concomitant separate evolutionary drivers of sex-related plumage in adult birds, we omitted data from specimens of unknown sex or recorded as immature. We did not select any patch type specifically, opting instead to encompass the full spectrum of avian plumage contrast. However, to avoid taxonomic imbalance in our study, we omitted a subset of some well-represented taxonomic groups—from studies that collected a large amount of data on a single group, for example, the estrildid finches [63]. Note, though, that all data in BirdColorBase are included in the supplementary spectrophotometry dataset for future use.

Because we were interested in the possible relationships between environment and plumage colour, particularly in visual communication, we measured plumage contrast using two approaches (A) bird vision models including the effects of ambient light, plumage reflectance and bird vision (following [27] when seen under open/cloudy light environments; open/cloudy light environments occur in all habitats, including at night when it is cloudy, or any time that there is no canopy [22]) and (B) bird vision models under the most likely light environment [22] based on a given bird species' main habitat and diel activity. For both approaches, we set the species eye type (Ultraviolet U or Violet V, following [27]) based on published data [23,27,28,68–70]; and phylogenetic relationships [27,71] using Jetz *et al*. [72]. Eye models follow that of Vorobyev & Osorio [73] as in Endler & Mielke [27]; see Renoult *et al*. [74] for a detailed discussion.

**Figure 1.**
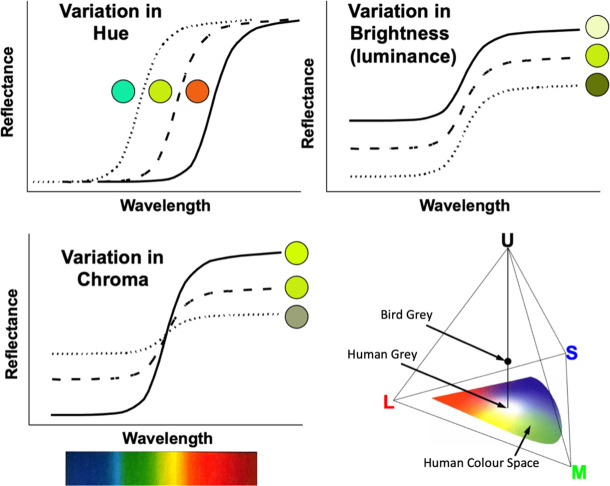
Physical and physiological meaning of hue, chroma, and brightness (luminance) shown by example spectral differences. Hue, chroma and brightness have a physical basis as shown in the three groups of spectra. ‘Brightness’ is a vague term which is often used for both total light intensity as well as chroma, or both. It is less ambiguous to use luminance. Any stimulus spectrum can be represented inside or on the surface of a tetrahedron. In the tetrahedron version of bird colour space (bottom right) the four avian cones are symbolized as U (ultraviolet-sensitive or UVS), S (short-wavelength or SWS), M (medium-wavelength or MWS) and L (long-wavelength or LWS). The tetrahedron (or the CIE colour space for humans) is independent of luminance. Perceived chroma is proportional to the distance from the grey point. Hue is measured by the angle from the origin grey point—for birds, there are two angles (as in latitude and longitude) but a single hue measure can be obtained from a two-dimensional colour space with axes defined by the difference between pairs of cones L–S and M–U. For details see [27].

For both approaches A and B, we used estimates of luminance, chroma and hue angle as in Endler & Mielke [27]. Luminance is a measure of the ‘brightness’ of a given colour patch. We do not use the term ‘brightness’ here because it is also often applied to high chroma colours. Chroma is a measure of how different a patch is from black/grey/white (flat spectra), i.e. the colour intensity or saturation. Hue is a measure of what parts of the spectrum are most intense in the patch (e.g. red, yellow or blue). For more information, see figure 1.

For both approaches, we estimated several measures of within-bird contrast using all adult spectra for a given species (i.e. across different studies/patches). All are related to contrast in bird plumages, and thus conspicuousness, as follows. Luminance: maximum (MaxL), minimum (MinL), difference (Diff), geometric mean (gMeanL) and geometric s.d. (gSDL; luminance is log-normally distributed). Chroma: mean (MeanCr) and s.d. (SDCr) and hue circular s.d. (SDHue) (table 1). Following Dunning *et al*. [43], we suggest that maxL may be predicted from the environment, but the other measures of within-bird contrast may also be related to the environment if they are important in intraspecific communication. For example, maxL is one measure of visual contrast. This contrast can either be within a given bird’s plumage or relative to the background or both. The difference between the lightest ('brightest’) and darkest patch (Diff) also affects contrast, and gmeanL and gSDL could also affect conspicuousness. Chroma and hue also affect conspicuousness and s.d. is a measure of the variation across the bird. The gmeanL and meanC are potential measures of possible contrast with the background depending upon average background coloration.

**Table 1.** Eight measures of three colour parameters (luminance, chroma and hue) used in this study, and their respective definitions. All are based upon bird eye models and calculated within species. See also figure 1 for heuristic interpretations of luminance, chroma and hue.

colour parameter	measure (abbreviation)	definition
*luminance*: how much light comes from a given colour patch.	maximum luminance (MaxL)	maximum luminance measured from a single patch, regardless of all other patches.
	minimum luminance (MinL)	minimum luminance measured from a single patch, regardless of all others.
	difference between MaxL & MinL (Diff)	the difference between the MaxL and MinL (i.e. the range between lightest and darkest plumage patch)
	mean luminance (gMeanL)	geometric mean luminance measured across patches. (as luminance is log-normally distributed) gMeanL is greatest in plumage made up of strongly reflective patches.
	s.d. of luminance (gSDL)	geometric s.d. of luminance measured across patches. gSDL is greatest where plumage spans a broader range of patch luminance.
*chroma*: intensity relative to black/grey/white	mean chroma (MeanCr)	mean chromatic intensity measured across patches. MeanChr is greatest in intensely saturated plumage, those that differ most from flat wavelength spectra (i.e. that differ most from black, grey, white).
	s.d. of chroma (SDCr)	s.d. of chromatic intensity across patches. SDCr is greatest where plumage spans a broader range of chroma.
*hue*: wavelength location of highest intensities (e.g. red/yellow/blue)	s.d. of Hue (SDHue)	s.d. of hue across patches measured as a circular SD since hues are distributed as angles over 360° (figure 1). SDHue is greatest in plumages with many differently coloured patches.

### (b) Behaviour and environmental traits

We extracted two discrete traits from AVONET (see [75] for details on how these scores were calculated) that capture the species-level light environment and viewing conditions: habitat openness and migratory tendency. Habitat openness (also known as ‘habitat density’) was scored in AVONET as one of three states for each species: 1 = dense habitat (e.g. the darkest parts of forests, woodland, scrub or thicket), strongly associated with the ‘forest shade’ ambient light environment [22]); 2 = semi-open habitat (e.g. scattered bushes, parkland or deciduous woodland), strongly associated with the ‘woodland shade’ environment [22]; and 3 = open habitat (e.g. desert, grassland, rocky, coastal, lacustrine or marine or spending most time above a vegetation canopy), strongly associated with the open/cloudy environment [22]. As score 1 was defined for the habitat with the highest density [75] and hence the darkest light environment, we prefer the term ‘openness’ over other possible synonyms for these scores.

Migratory tendency was also scored as one of three states for each species from AVONET: 1 = sedentary/non-migratory; 2 = partially migratory (e.g. those where migratory preference varies within a species, or where species migrate over short distances in response to seasonal change); and 3 = migratory (where the majority of the global population undertakes a long-distance migration).

We generated discrete diel activity scores using the *Handbook of Birds of the World* [76], supplemented by updated text from the online Birds of the World [77]. These states were defined as: 1 = nocturnal (species that primarily forage at night), 2 = mixed (species that have both diurnal and nocturnal behavioural elements, including crepuscular and cathemeral species as well as species exhibiting intraspecific variation in activity pattern), and 3 = diurnal (species that primarily forage during daylight). We aligned taxonomies with the AVONET database [75] and our database of spectral reflection measures.

### (c) Behavioural and environmental values based on light intensity

The original data on habitat openness, diel activity and migration tendency do not reflect quantitative differences in light environments. For example, light intensity varies log-normally, and eyes respond to the log of intensity [27]. Consequently, we performed an analysis where we accounted for light intensity using the mean habitat-specific ambient light intensity data from Endler [22]. The log_10_ mean light intensities of forest shade, woodland shade and open/cloudy are 1.1271, 1.5785 and 3.519, respectively [22], consequently, we coded habitat openness values as 2, 3 and 5 instead of the original habitat density ranks of 1, 2 and 3. The log_10_ means of night, crepuscular (early/late) and diurnal (mean of forest shade, woodland shade and open/cloudy) conditions are 0.1, 1.0 and 1.73 [22], consequently, we coded the diel activity values as 1, 10 and 17, respectively. For migration the log_10_ means for sedentary (mean of forest shade, woodland shade, open/cloudy), middle (mean of open/cloudy and sedentary mean) and migratory (open/cloudy) are 1.73, 2.396 and 3.062; consequently, we coded the migratory values as 3, 4 and 5, respectively. We then used integer equivalents of the same relative scaling because the original scores were simple ranks and we could not justify more than one significant digit. To do this, we multiplied by a constant and truncated the number (see supplementary files for code). For example, for migration, the log_10_ light intensities of 1.1271, 1.5785 and 3.519 are coded as 2, 3 and 5. For migratory species, we used 5 rather than 6 because we treated the open/cloudy habitat as a 5 in habitat openness and that is also the main light environment for migratory species.

### (d) Light environments for the eye calculations

Models of reception by eyes required information on the ambient light spectrum [22,27,73], which we approached in two ways (approaches A and B). For approach A, we used the open/cloudy light environment because this light spectrum is found in any habitat if there is no canopy, or when there is a canopy (shade) and the sky is covered with clouds [22]. This spectrum is also characteristic of moonlight [22]. For approach B, we estimated the most commonly used ambient light for each species based on its diel activity and habitat density. For crepuscular species, we set the ambient light to the early/late spectrum [22]. For the other species, we used habitat density to estimate the most common light environment [22]: dense = forest shade, semi-open = woodland shade and open = open/cloudy. All calculations included the von Kries correction; it accounts for correction to colour perception to maintain a constant in changing light environments, using the assigned light environment.

### (e) Statistical model specifications

We used the MCMCglmm R package [57] to run nine sets of hierarchical, Bayesian GLMMs with a Markov chain Monte Carlo extension in R version 4.3.1 [78]. The advantage of MCMCglmm is that allows statistical accounting for uncertainty within the phylogenetic relationships, as well as the construction of models with multiple random effects.

We ran two sets of analyses (A) contrast dependent upon avian eye models under open/cloudy light, and (B) contrast dependent upon avian eye models under the most appropriate (hereafter, natural) light environments for each species (see above). There were a total of four sets of analyses because we ran each set of analyses on two versions of the data: adult males and adult females.

For each model, we set three fixed effects: habitat openness, migratory behaviour and diel activity (see Material and methods above). To account for the effect of shared evolutionary history between species in our database and account for non-independence, we also included bootstrap replicates of the inverse of the genetic correlation matrix of 100 phylogenetic trees drawn from Jetz *et al*. [72]. We also included the sources of plumage measurements as random effects to account for variance in the sampling methodology.

We ran each model sequentially over each phylogenetic tree, with 22000 iterations per tree, a 2000 iteration burn-in period, and a 2000 iteration thinning interval, for a total of 10 samples per tree. Priors for the residual variance and the variance of the two random effects (phylogenetic signal and study origin) were drawn from relatively flat inverse-Wishart distributions with *V* = 1 and *ν* = 0.002, following the recommendations of Garamszegi & Mundry [79], while priors for the fixed effects were kept as the program default (diffuse normal priors with mean 0 and variance 10^10^).

We visually checked the posterior trace plots for all model outputs and ensured that autocorrelation was below 0.1 and effective sample sizes were between 1000 and 2000. We inferred a statistically significant association between plumage and behavioural traits where the 95% credible interval (CI) of the posterior distribution did not overlap zero. Finally, to account for false positives derived from running multiple tests, we ran a Benjamini–Hochberg false discovery rate (hereafter, FDR) [80] test in R, on the output pMCMCs (corrected results given in table 2; electronic supplementary material, table S2). A sample R script for the analysis is presented in electronic supplementary material, table S3.

**Figure 2.**
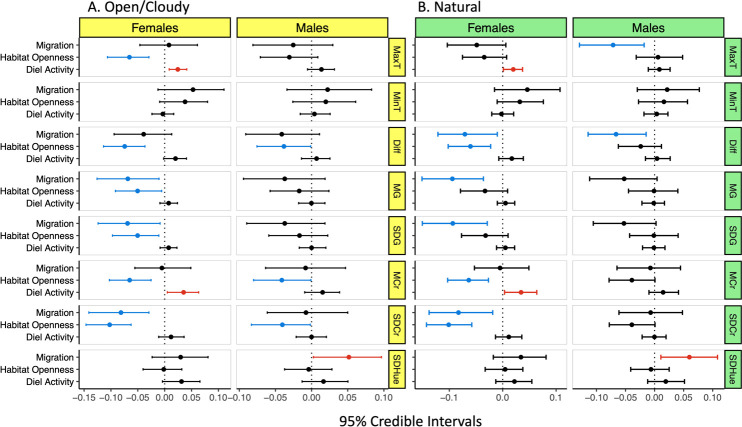
Forest diagram of MCMCglmm outputs, 95% credible intervals and posterior means for two light environments. Three behavioural measures (migration, habitat openness and diel activity), calculated for eight measures of plumage contrast (MaxT, MinT, Diff, MG, SDG, MCr, SDCr and SDHue—for acronym explanations, see table 1) and two light environments: (A) open/cloudy, which is present in all habitat types, and (B) natural (or appropriate), which aligns with habitat type. We inferred significance where the 95% credible intervals do not span 0. Where the credible interval is positive, we interpret a positive interaction between plumage contrast and behavioural trait (red), and where the credible interval is negative, we interpret a negative relationship (blue).

**Table 2.** pMCMC values, (A) without and (B) with false discovery rate (FDR) correction. pMCMC values are a posterior output of a Markov chain Monte Carlo simulation where lower values indicate stronger evidence that an effect differs from zero. we infer significance where pMCMC <0.05. Significant correlations between plumage contrast measures and the environment/ behavioural traits values are given with the direction of the effect (±). A negative correlation with habitat openness indicates more colour contrast in darker habitats. A positive correlation with migration indicates more colour contrast in migratory species. A positive interaction with diel activity indicates more contrast in nocturnal species. See table 1 for detailed definitions for each variable and figure 2 or the electronic supplementary material for full credible interval results.

(A) pMCMC									
trait	subset	maxL	minL	diff	gMeanL	gSDL	meanCr	SDCr	SDHue
habitat openness	females—natural light	0.092	0.148	**0.000 (−)**	0.147	0.147	**0.003 (−)**	**0.00 (−)**	0.829
	females— open/cloudy	**0.001 (−)**	0.107	**0.000 (−)**	**0.013 (−)**	**0.021 (−)**	**0.001 (−)**	**0.000 (−)**	0.937
	males—natural	0.771	0.458	0.219	0.961	0.94	0.06	0.051	0.718
	males—open/cloudy	0.119	0.359	**0.044 (−)**	0.412	0.426	**0.038 (−)**	**0.052 (−)**	0.835
migration	females—natural light	0.096	0.133	**0.012 (−)**	**0.001 (−)**	**0.002 (−)**	0.838	**0.00 (−)**	0.19
	females— open/cloudy	0.76	0.091	0.158	**0.024 (−)**	**0.018 (−)**	0.824	**0.002 (−)**	0.238
	males—natural	**0.012 (−)**	0.444	**0.010 (−)**	0.086	0.06	0.807	0.801	**0.02 (+)**
	males—open/cloudy	0.35	0.457	0.115	0.214	0.206	0.771	0.788	**0.038 (+)**
diel activity	females—natural light	**0.034 (+)**	0.834	0.128	0.495	0.501	**0.035 (+)**	0.337	0.182
	females— open/cloudy	**0.007 (+)**	0.733	0.068	0.327	0.337	**0.033 (+)**	0.31	0.077
	males—natural	0.353	0.692	0.696	0.879	0.883	0.234	0.997	0.228
	males—open/cloudy	0.165	0.652	0.469	0.972	0.985	0.216	0.996	0.301

(B) pMCMC after Benjamini–Hochberg FDR									
trait	subset	maxL	minL	diff	gMeanL	gSDL	meanCr	SDCr	SDHue
habitat openness	females—natural light	0.169	0.169	**0.002 (−)**	0.169	0.169	**0.008 (−)**	**0.002 (−)**	0.829
	females—open/cloudy	**0.002 (−)**	0.122	**0.002 (−)**	**0.021 (−)**	**0.028 (−)**	**0.002 (−)**	**0.002 (−)**	0.937
	males—natural	0.961	0.916	0.586	0.961	0.961	0.24	0.24	0.961
	males—open/cloudy	0.238	0.486	**0.14**	0.486	0.486	**0.14**	**0.14**	0.835
migration	females—natural light	0.154	0.177	**0.025 (−)**	**0.008 (−)**	**0.008 (−)**	0.838	**0.019 (−)**	0.217
	females—open/cloudy	0.824	0.183	0.254	**0.064**	**0.064**	0.824	**0.016 (−)**	0.318
	males—natural	**0.05 (−)**	0.592	**0.05 (−)**	0.138	0.12	0.807	0.807	**0.053 (+)**
	males—open/cloudy	0.56	0.609	0.428	0.428	0.428	0.788	0.788	**0.311**
diel activity	females—natural light	**0.143**	0.834	0.342	0.573	0.573	**0.143**	0.54	0.364
	females— open/cloudy	**0.058**	0.733	0.155	0.385	0.385	**0.134**	0.385	0.155
	males—natural	0.942	0.997	0.997	0.997	0.997	0.936	0.997	0.936
	males—open/cloudy	0.805	0.996	0.938	0.996	0.996	0.805	0.996	0.805

## 3. Results

The results of tests and FDR corrected tests are given in table 2 (and electronic supplementary material, S2), and 95% credible intervals in forest plots in figure 2. Results are arranged in two groups: adult males and adult females, and for the two light environments of open/cloudy and natural (appropriate for species-specific habitat type and activity times). The effect of traits on plumage contrast was strongest in females. Overall, habitat openness and migration resulted in significant effects over multiple contrast measures both achromatic (luminance) and chromatic (chroma), with no significant effect of diel activity except marginally in females in open/cloudy conditions.

### (a) Habitat openness

For females in open cloudy light, most contrast measures were significantly negative. Females in natural light showed the same significance patterns except the effects on maximum luminance and the mean and s.d. of luminance disappeared. These results remained after FDR tests. Generally, in females, higher contrast is associated with darker habitats (less openness), regardless of whether open/cloudy or natural light environment eye models were used. This relationship was found for both achromatic (the maximum, difference between minimum and maximum, mean and s.d. of luminance) and chromatic (mean and s.d. of chroma) measures (table 2A). For males in open/cloudy light, habitat openness predicted (CI did not overlap 0) the difference between maximum and minimum luminance measures and the mean and s.d. of chroma, but not in natural (appropriate to each species) light conditions. Effects were negative, indicating more open habitats are associated with lower contrast in males. However, following FDR tests the effects of habitat openness in males were lost (table 2A).

### (b) Migration

The effects of migration were strongly sex dependent (table 2B); females showed more and different contrast measure effects than males. Males showed a positive relationship between migration and the s.d. of hue, and only in natural light with negative relationships for maximum luminance, the difference between maximum and minimum luminance. Except for the relationships in natural light environments, effects in open light environments disappeared after FDR. Females showed negative coefficients in both light environments: minimum and the geometric mean of luminance and negative with geometric s.d. of luminance and s.d. of chroma in natural light environments and loosing of the difference of maximum and minimum luminance in open light environments. Effects were stronger in natural light. After FDR only negative relationships were found: with no change in natural light environments and only the s.d. of chroma in open light models. Note that this suggests that migratory males have higher hue contrast and females have lower luminance and chromatic contrast, and for both males and females, effects are stronger in natural (appropriate) light models.

### (c) Diel activity

Dial activity positively predicted maximum luminance and mean chroma for females in both open and appropriate light environments. However, following FDR tests, these effects disappeared.

## 4. Discussion

Our results demonstrate the importance of non-sexual drivers in the evolution of avian plumage contrast, including the role of avian vision systems and the relative light environment in these processes. Since Endler’s paper on forest light and colour was published in 1993 [22], many studies have demonstrated the importance of the light environment in the evolution of avian plumage complexity, often in specific study systems (including [9,36–39,43,47]). However, broad macroevolutionary approaches that account for variable light environments and in the context of bird-eye models, are still scarce.

We predicted that efficient communication arises from high visibility, thus that: (i) darker habitats (higher density [22]) should be associated with more contrasting coloration [10,22,47]; (ii) diel activity (activity within a day/night cycle) will correlate with plumage contrast, with more achromatic contrast in nocturnal and perhaps crepuscular species compared to diurnal species, as in Dunning *et al*. [43]; and (iii) species which migrate or otherwise form coherent groups should show higher contrast than those which are more sedentary because higher visibility makes coherent movements easier in flight and on the ground, in addition, migratory species have been associated with greater sexual selection driven by less time to mate [48]. We tested each in the context of differing light environments and bird-eye models. We found a clear relationship between plumage contrast and both light conditions, habitat openness and migratory tendency (table 2). Sex affects some contrast measures but not others, and the predictors are stronger and more diverse in females than males. In general, our prediction that habitat openness resulted in more plumage contrast is supported. Migratory species were less contrasting and with greater hue than non-migratory species, and diel activity did not predict plumage contrast in our broad study.

The effects of habitat openness and migration were significant under both eye-light models (bird vision models using open/cloudy or natural light), which include the effects of both spectral sensitivity and light adaptation via the von Kries correction [27,73]. This suggests that the avian vision-based contrast measures are reasonable and predictable from behaviour and viewing conditions. Both achromatic and chromatic contrast measures are predicted but differ in intensity and direction among males and females, especially for migration. The contrast measures based upon avian eye models showed greater contrast in darker environments particularly in females, supporting our hypothesis (significant coefficients negative; table 2A). The effect was slightly weaker under natural compared to open/cloudy light, possibly due to chromatic adaptation. The effect of ambient light was reversed for migration: that is, stronger contrast in natural light. This highlights the need for caution when interpreting the visual contrast of bird plumage without taking the light spectral conditions under which the plumage is seen. Light environments can change the perception of colour patterns [26,27,73,81], and this further demonstrates a role of the light environment in the evolution of avian plumage.

Females showed much stronger effects of habitat density than males. Specifically, habitat openness predicts a reduction in both luminance and chromatic contrast, particularly in females (table 2). Darker (less open) habitats favour brighter or more contrasting patches, and surprisingly, more chroma because colour is more difficult to detect in lower light levels. However, for diurnal species, there is enough light for colour vision [22]. All forms of communication may benefit from greater contrast in darker habitats. The differences between male and female contrast measures suggest that non-sexual communication and behavioural functions may be more important for those measures of contrast than straightforward sexual selection, and aligns with previous work suggesting that female plumage colours are more plastic than males [53–55]. This might result from strong selective roles played by the many non-sexual aspects of avian colour, such as species recognition, anti-predation, anti-eavesdropping, competition, habitat choice, thermoregulation and feather wear and many of these are relatively more important in females than males. In addition to non-sexual selection factors, the negative effect of migration on female contrast may reflect the predation danger to females by day at migratory stopover points, or in densely flocking species. This suggests that, although sexual selection favours sexual dimorphism, the effects of habitat and migration may partially counteract factors favouring sexual dimorphism. On the other hand, in some species males and females occupy slightly different habitats [82] or are active in different light environments [35], which may strengthen dimorphism.

We tested the hypothesis that diel activity should affect plumage contrast, because species that are active at night, or in dimly lit environments, should have more intense patches than those active during daylight [42,43,47]. We found no evidence to support our diel activity prediction, but this could at least be partially attributable to small evolutionary sample sizes; most species in our sample are diurnal. There is strong published evidence that light environments affect visual contrast during daylight [36,37,40,41], but this may be less important to nocturnal species, that exploit dark environments. Moreover, the effect of diel activity may have been overwhelmed by the joint evolutionary signatures of openness and migration, because although most migratory birds migrate at night [83], predator avoidance by day could be more important. It may also be the case that our data include samples of cryptic plumage, which has been otherwise associated with darker environments [10,41,84], but includes relatively few of the under-studied highly reflective patches that are often present but hidden until displayed in these species [42,43].

Although this hypothesis was quite speculative, we found a significant effect of migratory tendency on contrast measures (table 2B). This finding supports previous suggestions of lighter plumage in migratory birds, possibly driven by temperature regulation [16,50,85]. However, we found very different results in males and females: migratory males show more hue contrast and migratory females show less luminance and chroma contrast.

In females, high predation risk may be significantly associated with what happens at migratory resting sites or other forms of diurnal flocking [86,87]. This would favour less contrast in females than males. On average in males, migration is associated with more hue contrast and less luminance and chroma contrast. High male hue variation may be efficient in signalling in all light environments so perhaps males are the centres of focus in flock cohesion. The male results are surprising, as it might be easier for flock members to track achromatic than chromatic colours. This effect suggests an interaction with sexually selected pressures, or from the opposing relationships between colour and both sexual selection and predation (as in [35]). In addition, both migratory and nomadic species have short breeding seasons which suggests strong sexual selection, a phenomenon contradicted by monomorphism [88], but that could explain the divergent effects of sex. There is a complex relationship between patch shape, patch orientation and ability to track achromatic and chromatic patches [89], so sex- and species-specific colour patterns may create different visual effects depending upon how the birds themselves move and on the lighting and visual backgrounds. There may be a complex interaction between flock cohesion and sexual selection; differential behaviour in migratory species would repay further study.

There are multiple factors affecting avian plumage and visual contrast, and there are different combinations of biotic and abiotic factors affecting the achromatic and chromatic aspects of bird plumage. Multiple factors influence different plumage components in different ways [23], for example the appearance of an animal may be very different under chromatic and achromatic conditions [90]. We must not assume that the same kind of selection affects all plumage components and patterns across species or even within species. Our results suggest that we also need to consider non-sexual aspects of plumage as well as sex-associated differences in behaviour along with the effects of the light environment and avian vision. Finally, our findings open more questions for future study. We suggest that the stronger effects in females emphasize the need for further explicit scientific study of female plumage but also of sex-specific behaviours, the role and location of specific patch types, and habitat usage. It may also be the case that other, more specific behavioural and habitat preferences could predict plumage contrast, for example plumage contrast and background could be explored using vegetation cover indices or, species activity above and below the canopy.

Although our study was specific to birds, limited by the bird-specific eye models we used, further research could replicate what we have done on non-bird taxa, and between birds and their predators, where extensive data on colour vision and ambient light is available. Indeed, these principles are also demonstrated in mammals [33] albeit to a lesser extent [29], where sexual dichromatism is rare, but bare parts are sometimes used for signalling in a similar way to birds [91]. The colour of pelage (fur) and the bare parts of some primates is likely facilitated by the evolution of trichromatic vision [31,92], and more broadly, pelage colour is shaped by the light environment [30,32]. Our results underscore the importance of non-sexual drivers of avian plumage contrast, our study system, especially in the context of light environment. Future work on the evolution of bird plumage (or pelage) contrast should consider the light environment within which signals are emitted and received.

## Data Availability

We accessed data from published data repositories, AVONET and the BirdColorBase [67]. All of the processed data associated with this study are made available on Dryad [93]. Supplementary material is available online [94].
